# Repositioning the Alpha Cell in Postprandial Metabolism

**DOI:** 10.1210/endocr/bqaa169

**Published:** 2020-09-23

**Authors:** Kimberley El, Megan E Capozzi, Jonathan E Campbell

**Affiliations:** 1 Duke Molecular Physiology Institute, Duke University, Durham, North Carolina; 2 Department of Pharmacology and Cancer Biology, Duke University, Durham, North Carolina; 3 Department of Medicine, Division of Endocrinology, Duke University, Durham, North Carolina

**Keywords:** alpha-cell, beta-cell, glucagon, GLP-1, GIP, glucose homeostasis

## Abstract

Glucose homeostasis is maintained in large part due to the actions of the pancreatic islet hormones insulin and glucagon, secreted from β- and α-cells, respectively. The historical narrative positions these hormones in opposition, with insulin primarily responsible for glucose-lowering and glucagon-driving elevations in glucose. Recent progress in this area has revealed a more complex relationship between insulin and glucagon, highlighted by data demonstrating that α-cell input is essential for β-cell function and glucose homeostasis. Moreover, the common perception that glucagon levels decrease following a nutrient challenge is largely shaped by the inhibitory effects of glucose administration alone on the α-cell. Largely overlooked is that a mixed nutrient challenge, which is more representative of typical human feeding, actually stimulates glucagon secretion. Thus, postprandial metabolism is associated with elevations, not decreases, in α-cell activity. This review discusses the recent advances in our understanding of how α-cells regulate metabolism, with a particular focus on the postprandial state. We highlight α- to β-cell communication, a term that describes how α-cell input into β-cells is a critical axis that regulates insulin secretion and glucose homeostasis. Finally, we discuss the open questions that have the potential to advance this field and continue to evolve our understanding of the role that α-cells play in postprandial metabolism.

Glucagon, secreted by the pancreatic α-cells, has long been studied under the perception that it has the primary role of raising blood glucose to prevent hypoglycemia. A number of reviews of glucagon secretion have been published since the 1970s and resurfaced in the 2000s. The first reviews alluded to a role for glucagon in insulin secretion, but conclusions were centered on glucose-dependent changes in glucagon and insulin, highlighting paracrine actions to inhibit glucagon secretion.

The aim of this review is to present an alternative and updated perspective of how glucagon regulates metabolism, with a focus on the postprandial state, derived from recent evidence that the α-cell is essential for the regulation of insulin secretion and for glucose metabolism. This review will reference original and reviewed research that highlight the transitioning view of glucagon from its historical role as a counterregulatory hormone to having a more significant role in postprandial metabolism.

## The Incretin Effect

Much of nutrient absorption occurs in the proximal duodenum, which subtends the pancreas and receives bile from the liver (1). Enteroendocrine cells in the intestine, like K- and L-cells, sense nutrients in the lumen at different sections in the intestine: K-cells are predominantly located in the duodenum, while the highest density of L-cells are found in the ileum and colon (2, 3). Absorption of nutrients into the intestinal cells stimulates the release of glucose-dependent insulinotropic polypeptide (GIP) and glucagon-like peptide 1 (GLP-1) from K- and L-cells, respectively (4). Termed incretins, or *in*sulin se*cretins* (5), GIP and GLP-1 fulfill this definition by relaying nutrient intake to enhanced insulin secretion through endocrine actions mediated by incretin receptors located on β-cells. The GIP receptor (GIPR) and GLP-1 receptor (GLP-1R) are class B G-protein coupled receptors that potentiate glucose-stimulated insulin secretion in β-cells. Both GIP and GLP-1 require elevated glucose to potentiate insulin secretion, and have little to no action on insulin secretion at low glucose (6, 7). The dependency on elevated glucose allows the incretin axis to fine-tune insulin secretion to reflect the magnitude of nutrient ingestion. In other words, a larger meal provokes greater insulin secretion to maintain euglycemia in large part due to the incretin axis. The incretin effect is defined as the greater insulin secretory response to glucose administered orally than to glucose given intravenously (8), which encapsulates the importance of these hormones for postprandial glucose metabolism.

A number of recent observations have challenged the conventional model of the incretin axis. It is clear that both the GIPR and GLP-1R are the primary mediators of the incretin action in β-cells. Glucose-dependent insulinotropic polypeptide continues to be considered a gut-derived peptide that behaves in an endocrine manner to stimulate the GIPR on β-cells, although there is some evidence of GIP production locally in the islets (9, 10). On the other hand, the source of the ligand for the GLP-1R is less clear (11). The location of GLP-1 secreting L-cells in the distal gut, far from nutrient absorption, suggests its function in the sensing and responding to nutrients is secondary and that GIP may be the primary gut-derived incretin. However, a number of mechanisms have been proposed to enable the secretion of GLP-1 through signals originating in the proximal gut, including a neuroendocrine loop mediated by the vagus nerve and GIP (12–14), demonstrating that nutrients do not need to reach the distal gut in order to stimulate GLP-1 secretion. Still, nutrient intake stimulates an 8- to 10-fold increase in GIP, but only a ~2-fold increase in GLP-1 (11). To this end, it has been shown that intestinal preproglucagon (*Gcg*) expression, which is responsible for the production of GLP-1, is dispensable for the hyperglycemic effects of GLP-1R antagonism in mice (15), suggesting that gut-derived GLP-1 has minimal activity at the GLP-1R in β-cells. A separate set of studies demonstrated that loss of *Gcg* in the gut does not impair oral glucose-stimulated insulin secretion in mice (16), reinforcing that gut-derived GLP-1 minimally interacts with the β-cell GLP-1R. The modestly impaired glucose tolerance in this model was attributed to elevated gastric emptying, supporting a role for gut-derived GLP-1 in postprandial glucose metabolism, just not at the level of the islet. These recent papers raise the question of what ligand is responsible for the insulinotropic effects of β-cell GLP-1R signaling. The hyperglycemic effects of GLP-1R antagonism were present in mice with select expression of *Gcg* in α-cells (15), suggesting that the β-cell GLP-1R is stimulated primarily from paracrine factors originating in the islet. Conventionally, the α-cell processes preproglucagon specifically to generate glucagon, not GLP-1 (17). However, there is evidence showing the potential for stress factors such as streptozotocin or interleukin-6 to induce changes in the α-cell that enable differential processing of preproglucagon to yield GLP-1 (18–20). These data postulate that the primary source of GLP-1 that engages the β-cell is from the α-cell, not the gut. Complicating this conclusion is the observation that glucagon is also a ligand for the GLP-1R (21), although with less activity than GLP-1. In mouse islets, glucagon is secreted at levels that are 100–300x more than GLP-1, but GLP-1 is 100–300x more potent at stimulating insulin secretion compared with glucagon (6). Thus, more work is needed to determine if and when GLP-1 or glucagon acts as the primary ligand for the β-cell GLP-1R. Still, the recent observations supporting the notion that the α-cell is the primary source of this ligand has forced a reconsideration of the incretin axis. It seems that GIP is the primary incretin derived from the intestine that signals to the islets through the GIPR, whereas the factors that stimulate GLP-1R signaling come from the α-cell.

## What Do We Actually Eat?

**Figure 1.**
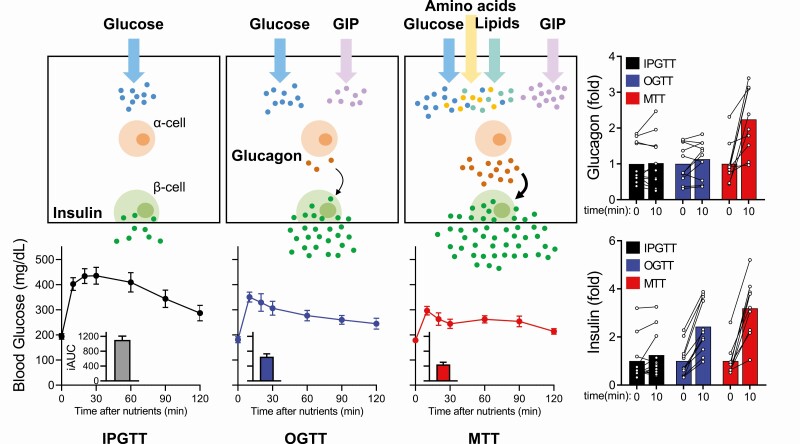
A comparison of the glucagon, insulin, and glycemic response to various nutrient challenges. Glucagon and insulin secretion increase from IPGTT → OGTT → MTT, while the glycemic response decreases from IPGTT → OGTT → MTT. All 3 challenges contain the same glucose load. Abbreviations: GIP, glucose-dependent insulinotropic peptide; IPGTT, intraperitoneal glucose tolerance test; OGTT, oral glucose tolerance test; MTT, mixed meal tolerance test.

Diabetes is defined as inappropriately elevated blood glucose due to insufficient insulin production. The gold-standard to diagnose type 2 diabetes (T2D) in patients is the oral glucose tolerance test (OGTT), where blood glucose levels are monitored in response to ingested glucose. The current concept of the incretin axis was originally defined by the elevated insulin response to orally ingested glucose versus an isoglycemic load given intravenously (5). The insulinotropic actions of incretins in β-cells are described as glucose-dependent, and much of the research that has led to the development of antihyperglycemic drugs, including incretin mimetics, has focused on these glucose-lowering properties. As such, the diabetes field has comfortably adapted to using glucose as a surrogate for nutrient intake. Yet, typical human feeding does not consist of consuming strictly carbohydrates as the primary source of nutrients. In fact, a mixed nutrient intake versus a pure carbohydrate stimulus produces dramatically different effects on insulin secretion and the subsequent glycemic excursion. As stated earlier, glucose given orally versus parentally produces a much more robust insulin secretion effect, generally attributed to the incretin axis. When the same bolus of glucose is given intravenously versus orally, the glycemic excursion in response to oral glucose is much lower. This is in part due to the delay caused by gastric emptying and intestinal absorption of glucose, but oral glucose also leads to increased insulin secretion. However, when isocaloric amounts of glucose are combined with other macronutrients, typically in the form of a mixed meal challenge, there is a further enhancement of insulin secretion and glycemic lowering (22). In other words, a meal challenge provides a more robust stimulus on the β-cell to enhance insulin secretion compared to oral glucose alone, even though the carbohydrate load is the same (Fig. 1).

What factors contribute to enhanced insulin secretion and glucose lowering when additional nutrients are combined with glucose? Incretins are secreted in response to lipids and amino acids, as well as to glucose. Introducing amino acids during a mixed meal tolerance test (MTT) induces markedly more GIP than an OGTT, whereas GLP-1 levels are increased marginally (23, 24). Intuitively, studies of incretin action in the islet have focused on the β-cell and insulin secretion, but incretins also affect the α-cell and glucagon secretion. Glucagon-like peptide 1 inhibits glucagon secretion likely through indirect effects on the β-cell and δ-cell from intra-islet signals (25, 26), although recently it was suggested that GLP-1 suppressed glucagon directly through GLP-1R on the murine α-cell and that GLP-1 can also stimulate glucagon secretion at low glucose levels (27). Yet, the observation that GLP-1 receptors are present on the α-cell is unsubstantiated by most studies in both rodents and humans (28–31), and most recently by islet cell populations sorted into enriched individual cell types by fluorescence-activated cell sorting (FACS) using a validated GLP-1R antibody, and then analyzed by reverse transcription polymerase chain reaction (RT-PCR) (32). Additionally, while both the GLP-1R and GIPR are expressed in β-cells, only the GIPR is expressed in α-cells (33). GIPR activation in α-cells stimulates glucagon secretion, but how this directly contributes to β-cell function and insulin secretion has not been tested.

The GIPR varies with different nutrient stimuli. Generally, administration of oral glucose induces a 2-fold increase in GIP levels in humans (34–39), while the levels of GIP are increased by 3-fold in response to lipid intake (40–44) and 4-fold in response to protein intake (24, 43, 45, 46). Thus, the enhanced β-cell effect of a mixed nutrient stimuli could be explained in part by a more robust activation of the incretin axis. Furthermore, both lipids and amino acids have direct effects on β-cell function that can serve to amplify insulin secretion independent of the incretin axis (47). However, central to this review is the stark difference on the α-cell invoked by glucose alone versus a mixed-nutrient stimulus (Fig. 1). A glucose challenge exerts a negative effect on α-cell function and reduces glucagon secretion (48). This is more potently seen with intraperitoneal/intravenous glucose compared with oral glucose, possibly due to the effects of GIP on the α-cell during an OGTT (49). Glucose stimulus alone activates both the β-cell and δ-cell, with a subsequent elevation in the inhibitory tone on the α-cell and a decrease in glucagon secretion (50). Loss of β-cell function in diabetes decreases this inhibitory tone, and the glucose stimulation is less effective at reducing glucagon levels (51). Because of this, T2D is often described as presenting with inappropriately high glucagon levels following a glucose tolerance test (49). However, a mixed nutrient stimulus incorporates potent activators of the α-cell, including glucagonotropic amino acids and fatty acids (24), overcoming the inhibitory paracrine actions and leading to an increase in glucagon secretion (52). Interestingly, direct glucagonotropic effects of GIP have been reported during hypoglycemia and euglycemia, but not hyperglycemia (53), unless there was underlying metabolic disease (54). The physiological relevance of GIP-stimulated glucagon during hypoglycemia is unclear, since GIP is secreted in response to nutrients and is expected to be at low levels during hypoglycemia. In response to a typical meal, α-cell function is enhanced in the postprandial state and glucagon levels become elevated. Thus, if stimulation of glucagon secretion in the postprandial state is the expected physiological response, how can glucagon levels be inappropriately elevated?

## Stimulation of Glucagon Secretion

What are the factors that lead to a rise in glucagon secretion following a mixed nutrient stimulus? Isoglycemic intravenous glucose lowers plasma glucagon more robustly than oral glucose in healthy subjects, a phenomenon that is magnified in patients with T2D, potentially due to an enhancement in the factors that limit glucagon suppression during an oral glucose challenge (49). This difference between intravenous and oral glucose suggests that a gut-derived factor counters the inhibitory actions of glucose alone on the α-cell, pointing to a potential role of the incretin axis. GLP-1R agonists are potent inhibitors of glucagon secretion (55), while GIP stimulates glucagon secretion (56). Could the stimulation of GIP by oral glucose be the factor that limits the suppression of glucagon secretion during an oral glucose challenge compared with an intravenous glucose challenge? Studies directly testing this hypothesis with loss-of-function interventions for the GIPR are needed to answer this question. In healthy humans given a mixed nutrient meal, the GIPR antagonists GIP(3–30) led to a modest decrease in glucagon levels that were not statistically different from control (57). However, GIP(3–30) also significantly elevated the glycemic excursion and lowered the insulin response during this test, making it difficult to assess the direct effects of GIPR antagonism on the α-cell.

Compared to an OGTT, a mixed nutrient meal produced a greater response in the α-cell, leading to enhanced glucagon secretion. It is possible that the presence of additional macronutrients in the gut lumen leads to a greater GIP response and a greater stimulation of the α-cell. As stated earlier, amino acids and lipids are potent regulators of GIP secretion compared with glucose alone. However, there is also evidence that, unlike glucose that confers an inhibitory signaling on α-cells, amino acids and lipids stimulate glucagon secretion. Administration of intralipid in healthy humans robustly stimulated glucagon secretion (44). Interestingly, when comparing orally versus intravenously administered intralipid, at doses that produce the same concentrations of circulating triglycerides, oral administration produced a greater rise in glucagon, GIP, and GLP-1 levels, hinting at an interaction between gut peptides and lipids on α-cell function. The mechanism by which lipids enhance glucagon secretion is still unclear. G protein-coupled receptors (GPCRs) that bind fatty acids are expressed in α-cells (33), and agonism of Gpr119 (58), Gpr120 (59), and Gpr40 (60) have been shown to induce glucagon secretion. On the other hand, the intracellular metabolism of lipids and the generation of adenosine triphosphate (ATP) has also been shown to drive glucagon secretion (61). Many of these studies have been conducted in the context of low glucose, where enhancing α-cell function is anticipated to counter hypoglycemia. Thus, the contribution of lipids to α-cell function in the postprandial state remains less clear.

Amino acids have long been known to potently induce glucagon secretion, although not all amino acids are equal in their glucagonotropic activities (62, 63). Historically, arginine has been the most commonly used amino acid to induce both insulin and glucagon secretion; however, it is commonly used at concentrations that far exceed physiological circulating levels. Indeed, most studies that have defined the glucagonotropic activities of individual amino acids have used supraphysiological concentrations (62, 63), which limit the extension of these findings to the context of a postprandial setting. Nonetheless, it is generally accepted that branch chain amino acids (leucine, isoleucine, valine) do not stimulate the α-cell (62, 64), while arginine, alanine, and glycine do when used at physiological concentrations. The factors that mediate the differential glucagonotropic properties of specific amino acids remains unresolved (65). In particular, alanine potently stimulates glucagon secretion at concentrations as low as 0.5 mM (66), which aligns with plasma concentrations of alanine (67). The intimate relationship between amino acids and the α-cell is highlighted by the liver–α-cell axis (68). This axis was originally identified by the substantial α-cell hyperplasia that occurs following interruption of glucagon signaling in hepatocytes (69), which was subsequently attributed to the dramatic rise in amino acids that were no longer being catabolized due to the lack of hepatic glucagon signaling (67). This phenomenon strikingly resembles the degree of β-cell hyperplasia ensuing after chronically blocking the insulin receptor and inducing hyperglycemia (70). This strong relationship between amino acids and the α-cell has spurred the idea that the primary role for α-cell is the regulation of amino acid metabolism, not glucose metabolism (65). Interestingly, perifusion of mouse or human islets with the amino acids arginine, glutamine, or alanine produces a biphasic pattern of glucagon secretion, denoted by a strong first phase and followed by a sustained second phase at a lower rate, remarkably similar to glucose-stimulated insulin secretion (6, 71). Could amino acids be the major secretagogue that stimulates glucagon secretion, very similar to the primacy of glucose for insulin secretion? Directly testing this is difficult given the numerous and promiscuous transporters for amino acids. However, a reduction in mammalian target of rapamycin (mTOR) signaling in α-cells, which is a central node for intracellular amino acid metabolism (72), impairs glucagon secretion and strongly implicates the importance of amino acids for α-cell function (73). Finally, the reduction in glycemic excursion and enhanced insulin and glucagon secretion produced by a mixed nutrient challenge, relative to an intraperitoneal glucose tolerance test, can be replicated by the addition of just alanine to an intraperitoneal glucose tolerance test (71). Therefore, if glucose is the primary fuel for the β-cell, it seems plausible that amino acids are the primary fuel for α-cells. Moreover, activation of the α-cell by amino acids is necessary for insulin secretion, a term we have called α- to β-cell communication (6, 52, 71).

## α- to β-Cell Communication in Fed Conditions

Glucagon has long been known to stimulate insulin secretion, despite glucagon being considered a counterregulatory hormone that opposes insulin action (74, 75). In patients given an intravenous bolus of glucagon, insulin levels rose within a minute (74). Moreover, a dose of glucagon given in a hyperglycemic state led to greater insulin secretion (74). This demonstrated a glucose-dependent insulinotropic action of glucagon, similar to that observed with incretins. The proximity of α-cells and β-cells positions β-cells to respond to the large paracrine signal from islet α-cells.

α- to β-cell communication and the subsequent enhancement of insulin secretion are lost in mice with deletion of proglucagon peptides in the α-cell or if the receptors for proglucagon peptides (GLP-1R and glucagon receptor [GCGR]) are silenced (6, 15, 71). However, these experiments do not clarify which proglucagon peptide is responsible for α- to β-cell communication. On one hand, some studies argue that GLP-1 may be produced in the α-cell in meaningful amounts to affect insulin secretion and thereby systemic glycemia (15, 76, 77). Furthermore, the production of GLP-1 may be amplified during metabolic stress through increased PC1/3 expression in α-cells (18, 78) and GLP-1 is a more potent insulin secretegogue than glucagon (6, 79, 80). However, there remains an ongoing debate on whether α-cells produce bona fide, bioactive GLP-1. First, many assays are unable to distinguish the bioactive form of GLP-1 (GLP-1 (7-36)) from the inactive 9–36 or 1–36 isoforms, which have been argued to be the predominant forms of GLP-1 from α-cells (81). A mouse model of α-cell deletion of PC1/3 was used to argue for GLP-1 production in α-cells (78); however, the validity of the knockout was incompletely described and the reduction in measured GLP-1 was modest. Deletion of intestinal *Gcg* drastically reduced plasma levels of active GLP-1, but only modestly reduced total GLP-1 levels (16). This suggests that α-cells do not contribute meaningfully to circulating concentrations of active GLP-1. Finally, further complicating the debate is the recent observations that glucagon stimulates insulin secretion primarily through the GLP-1R (6, 71, 81). Although some activity is noted at the GCGR, the majority of glucagon-stimulated insulin secretion is mitigated by antagonism or deletion of the GLP-1R. This complicates the dissociation of glucagon versus GLP-1 activity in β-cells, as both ligands engage the same receptor. Re-examining the previous work utilizing Ex9 to argue for α-cell GLP-1 (15) dampens the enthusiasm for this evidence, as all of these data could be attributed to glucagon. Therefore, α- to β-cell communication is attributed broadly to proglucagon products until further studies can specify the extent to which glucagon or GLP-1 uniquely contribute to intra-islet communication.

β-cells express both the GLP-1R and GCGR, which utilize cyclic adenosine monophosphate (cAMP) as a secondary messenger to stimulate insulin secretion (6). The GCGR is also highly expressed in the liver (68), while the GLP-1R is not (82). While it is clear that glucagon signals through the hepatic GCGR to exert effects on glycemia, the role of the GCGR in the β-cell is more complex. GCGR possesses a high degree of homology with GIPR and GLP-1R (83), congruent with its insulinotropic actions. However, an early study using the GCGR inhibitor, des-His^1^-[Glu^9^]glucagon-amide, showed that unlike hepatocytes, blockade of GCGR in rat β-cells had little effect on glucagon-induced cAMP induction (21). However, the GLP-1R inhibitor, Exendin 9 (Ex9), partially blocked glucagon-stimulated insulin secretion. These findings have since been corroborated (6, 81, 84, 85), and found to be more pronounced with physiological levels of glucagon stimulation in mouse pancreas (81), mouse islets (6, 85), and human islets (6, 84). These studies showed that both GCGR and GLP-1R are important for glucagon-stimulated insulin secretion, but that GLP-1R predominates for the insulinotropic actions. Moreover, recent studies have shown the crucial role for the α-cell in determining the tone of islet insulin secretion (6, 84); the dynamics of insulin secretion are maintained with loss of proglucagon products, but the magnitude of insulin secretion is substantially dampened. It is now appreciated that in isolated islets, proglucagon tone is necessary for the full extent of nutrient-stimulated insulin secretion.

The results of ex vivo studies have been supported by several in vivo studies using a range of techniques to demonstrate the importance of α- to β-cell communication. Interestingly, it was recently shown that islet grafts carry the glycemic setpoint in a species-dependent manner, and that this relies on glucagon content of the donor (84). In these studies, immunodeficient mice made diabetic with streptozotocin received islet xenografts in the eye or kidney that generated a glycemic set point in line with the species of the transplant. Importantly, human-specific glucagon receptor blockade by the antagonist L-168,049 in mice with human islet xenografts raised systemic glycemia (84). Further support for the importance of α- to β-cell communication is observed in mice lacking the GLP-1R and receiving glucagon receptor antagonist (GRA). Glucagon receptor antagonist consistently lower glycemia, which has primarily been attributed to a reduction in hepatic glucose output. A limitation to clinical GRA use, however, is that it leads to significant α-cell hyperplasia and elevation of circulating glucagon. However, with the perspective that glucagon can act through the β-cell GLP-1R to lower glycemia, it would be expected that large elevations in glucagon could lead to significant increases in insulin that may be equally important for lowering glycemia. Indeed, pre-treatment of mice given a GRA with GLP-1R antagonist Ex9 reduced efficacy of a GRA to lower glucose (86). Furthermore, the hypoglycemic phenotype of *Gcgr*^-/-^ mice in response to a glucose tolerance test is absent in *Gcgr:Glp1r*^*-/-*^ mice (87). The hepatic contribution of glucagon signaling clearly contributes to the lowered glycemic phenotype in models with loss of function for glucagon signaling, evident by the phenotype of *Gcg*^*-/-*^ mice that lack glucagon and GLP-1 (15). However, the enhanced α- to β-cell communication that results from blockade of glucagon signaling is an underappreciated contributor that warrants further investigation (80).

Recent work demonstrates the importance of the nutritional state in dictating the glycemic impact of glucagon. When 1 mg/kg glucagon was administered to fed mice, there was a significant induction of insulin that led to an overall lowering of glycemia (71). Alanine produced a similar effect to lower glycemia while increasing circulating glucagon (71), demonstrating that the glucose-lowering effects can be achieved by endogenous α-cell activity. Moreover, the glucose lowering effect of exogenous or endogenous glucagon activity requires β-cells and insulin secretion, as β-cell–specific deletion of *Glp1r* and *Gcgr* prevented glucose lowering in response to either exogenous glucagon or alanine (71). Additionally, an approach utilizing a Gi-coupled designer receptor exclusively activated by designer drugs (to lower glucagon secretion from the α-cell showed that a significant loss of plasma glucagon levels coincided with a drop in plasma insulin and glucose intolerance in an intraperitoneal glucose tolerance test (IPGTT) (85). We have also recently validated that the insulinotropic actions of glucagon prevail using ketone production from the liver as an output of hormone action (88). Indeed, treatment with exogenous glucagon significantly lowered ketone production within 20 minutes, in line with insulin action on ketosis (88). The ability for glucagon to stimulate ketone production only prevailed when insulin secretion was completely removed with the combination of streptozotocin, to delete the majority of β-cells, and treatment with an insulin receptor antagonist. Overall, these studies demonstrate that α- to β-cell communication is necessary for setting the tone of insulin secretion and overall glycemia in normal physiology.

## α- to Hepatocyte Communication in Fed Conditions

The canonical view of glucagon focuses on its hepatic actions as a hypoglycemia-responsive hormone functioning in the absence of ingested mixed nutrients. Indeed, glucagon potently activates glycogenolysis and gluconeogenesis through activation of GCGR and cAMP/protein kinase A (PKA) signaling in fasting conditions. Yet, glucagon levels actually decrease following prolonged fasting (>3 days) (89), and the addition of glucagon to patients during a prolonged fast did not elevate glycemia (90). These findings question the notion that glucagon’s primary role is to coordinate the glycemic responses to a prolonged fast. However, it is important to note that prolonged fasting significantly reduces hepatic glycogen content, potentially minimizing the ability for glucagon to enhance hepatic glucose output. Nonetheless, these data show that additional mechanisms other than glucagon maintain glycemic levels in the context of sustained fasting. In fact, an overnight fast in *Gcg*^*-/-*^ mice produces the same reduction of glycemia as control mice (88). On the other hand, several recent studies in liver suggest a role for glucagon that extend beyond the prevention of hypoglycemia and into the control of nutrient metabolism during the fed state.

The culmination of early landmark studies and recent work supports the notion that glucagon is a master regulator of amino acid metabolism. Amino acids provide a carbon source for several catabolic processes, while the nitrogen backbone is removed through urea. Maintenance of amino acid levels and, similarly, ureagenesis are processes highly responsive to glucagon. In both human studies (91–93) and animal models (94–97), glucagon treatment drastically modulates urea synthesis and amino acid levels. Furthermore, infusion of glucagon above basal levels, with glucose, amino acids, and insulin to mimic a postprandial state, promoted increased amino acid utilization in catheterized dogs (96). Studies using GCGR deletion or blockade in mice have profound increases in circulating amino acids, eliciting α-cell hyperplasia and hyperglucagonemia (69, 98–101). Conversely, patients with glucagonomas, characterized by hyperglucagonemia, have substantially elevated ureagenesis and lowered circulating amino acids. In fact, glucagonomas present with a skin rash, necrolytic migratory erythema, which is a direct result of severe hypoaminoacidemia (102). These findings demonstrate a critical liver– α-cell axis communicated through circulating amino acid levels. Indeed, the drastic changes in amino acid levels following glucagon manipulation support the notion that glucagon is a master regulator of hepatic protein metabolism. As such, glucagon’s hyperglycemic actions may be a secondary mechanism to promote conversion of amino acids to glucose in order to maintain appropriate circulating concentrations.

In a mixed nutrient meal, glucagon responds to amino acid levels to regulate protein metabolism, while insulin is secreted to manage carbohydrate metabolism. In this case of normal physiology in response to meal ingestion, the various nutrients would cause glucagon and insulin secretion into the portal circulation simultaneously. However, the conventional understanding of glucagon being a hypoglycemic hormone has limited the study of glucagon and insulin costimulation of hepatocytes in a prandial state. While there have been studies of bihormonal actions in the liver, they have largely been studied in the context of glucagon rescue for insulin-induced hypoglycemia, which would not mimic the secretory dynamics of the 2 hormones.

There is some evidence to suggest that glucagon and insulin have cooperative actions in hepatocytes. Interestingly, pretreating mice with a glucagon receptor agonist led to enhanced glucose lowering during a subsequent intraperitoneal glucose tolerance test (103). This effect was attributed in part to increased hepatic insulin sensitivity denoted by increased phosphorylation of AKT (pAKT473), an integral step of hepatic insulin signaling (103). While it is possible that glucagon agonism stimulated insulin secretion in β-cells, this effect has recapitulated in isolated hepatocytes, suggesting a specific action of glucagon in the liver. Notably, liver-specific GCGR deletion mitigated insulin-induced pAKT in hepatocyte cultures (103), suggesting a direct cross-talk of glucagon and insulin at the liver that remains undefined. A recent study using whey protein feeding in healthy human subjects reported induction of both insulin and glucagon, along with increased endogenous glucose production (104). This finding suggests that the glycemic lowering that results from the combination of insulin and glucagon is not only because insulin overcomes the ability for glucagon to stimulate hepatic glucose production. The hepatic signature of insulin and glucagon costimulation remains an open question in the field, with important implications for understanding the hormonal control of glucose homeostasis.

## Conclusion

The recent progress in our understanding of how the α-cell contributes to postprandial metabolic homeostasis has forced a reconsideration for the role of proglucagon peptides in response to food intake. While the GLP-1R in β-cells continues to be a key regulator of insulin secretion and glucose homeostasis, it is less clear what activates this receptor. The emerging evidence suggests that gut-derived GLP-1 does not interact with the GLP-1R, but rather that GLP-1 might originate from the α-cell. Alternatively, glucagon enhances insulin secretion via the β-cell GLP-1R, questioning the need for GLP-1 as a β-cell ligand altogether. Future studies are needed to delineate which hormone is primarily responsible for governing insulin secretion, and whether this changes with the progression of metabolic dysfunction. Still, the concept that the α-cell is required for β-cell function has re-oriented the relationship between α- and β-cells. Indeed, rather than carrying out opposing roles in the balance of glucose homeostasis, α- and β-cells cooperate in a postprandial setting to optimally regulate the metabolic control of nutrients.

The question remains as to why glucagon is abnormally elevated in patients with diabetes and does not lower in response to a carbohydrate load in these patients. We believe that recent data suggests that glucagon elevation is an adaptive reaction as opposed to an early causative step in hyperglycemia, similar to the accepted view that the hyperinsulinemia induced by insulin resistance is an adaptive function of β-cells. The root cause driving hyperglucagonemia remains unknown. Candidate mechanisms include a reduction in inhibitory tone as β-cell function diminishes, α-cell hyperplasia/hypertrophy in response to metabolic stress, enhanced GIP levels that increase α-cell activity, and an increase in amino acid tone on the α-cell due to either enhanced amino acid flux or decreased amino acid catabolism.

The α-cell is a powerful and central regulator of metabolic homeostasis. To fully appreciate the role that α-cell products have on postprandial nutrient metabolism, the field has begun to expand beyond the role of glucagon as a counterregulatory hormone that works to limit hypoglycemia. Furthermore, expanding these investigations beyond glucose metabolism enables a better appreciation for the additional mechanisms that govern metabolic control in a postprandial state. Understanding these mechanisms can better identify the compliment of pathophysiological complications that ultimately drive the metabolic dysfunction that leads to diabetes. By incorporating the α-cell into the equation, the field is moving towards a more complete picture of metabolic homeostasis.

## Data Availability

Data sharing is not applicable to this article, as no datasets were generated or analyzed during the current study.

## Additional Information

***Disclosure Summary:*** K.E. and M.E.C. are funded by fellowships from the National Institutes of Health (NIH) (T32 DK007012, F32 DK116542). J.E.C. is funded by the American Diabetes Association (ADA) (1-18-JDF-017) and NIH (R01 DK123075, R01 DK125353) and is a Borden Scholar. The Campbell lab receives funding for basic science projects unrelated to this work from Eli Lilly and Novo Nordisk. J.E.C. has received a speaker honorarium from Merck in the past 12 months.
